# Nuclear Receptors as Multiple Regulators of NLRP3 Inflammasome Function

**DOI:** 10.3389/fimmu.2021.630569

**Published:** 2021-02-26

**Authors:** Ahmad Alatshan, Szilvia Benkő

**Affiliations:** ^1^Departments of Physiology, Faculty of Medicine, University of Debrecen, Debrecen, Hungary; ^2^Doctoral School of Molecular Cellular and Immune Biology, Faculty of Medicine, University of Debrecen, Debrecen, Hungary

**Keywords:** inflammasome, NLRP3, IL-1β, signaling, PPAR, LXR, PXR, metabolism

## Abstract

Nuclear receptors are important bridges between lipid signaling molecules and transcription responses. Beside their role in several developmental and physiological processes, many of these receptors have been shown to regulate and determine the fate of immune cells, and the outcome of immune responses under physiological and pathological conditions. While NLRP3 inflammasome is assumed as key regulator for innate and adaptive immune responses, and has been associated with various pathological events, the precise impact of the nuclear receptors on the function of inflammasome is hardly investigated. A wide variety of factors and conditions have been identified as modulators of NLRP3 inflammasome activation, and at the same time, many of the nuclear receptors are known to regulate, and interact with these factors, including cellular metabolism and various signaling pathways. Nuclear receptors are in the focus of many researches, as these receptors are easy to manipulate by lipid soluble molecules. Importantly, nuclear receptors mediate regulatory mechanisms at multiple levels: not only at transcription level, but also in the cytosol via non-genomic effects. Their importance is also reflected by the numerous approved drugs that have been developed in the past decade to specifically target nuclear receptors subtypes. Researches aiming to delineate mechanisms that regulate NLRP3 inflammasome activation draw a wide range of attention due to their unquestionable importance in infectious and sterile inflammatory conditions. In this review, we provide an overview of current reports and knowledge about NLRP3 inflammasome regulation from the perspective of nuclear receptors, in order to bring new insight to the potentially therapeutic aspect in targeting NLRP3 inflammasome and NLRP3 inflammasome-associated diseases.

## Introduction

### The Nuclear Receptors

Nuclear receptors (NRs) are ligand-dependent transcription factors that regulate numerous physiological mechanisms, including development, differentiation, metabolism and immune functions (1, 2). Generally, nuclear receptors are activated by endogenous or exogenous small lipophilic molecules to control the transcription of a complex network of gene sets for a targeted function (3). In addition to their role as transactivators or transrepressors (genetic level), several NRs may initiate a processes of histone modifications and chromatin remodeling (epigenetic level). Furthermore, increasing number of evidence shows that they are involved in various intracellular, non-genomic functions; such as the modulation of signal transduction pathways and cell membrane receptors activity (4, 5).

**Figure 1:**
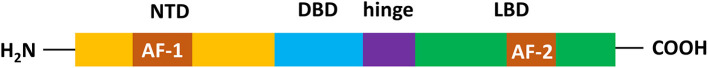
Representation of the domains structure of the nuclear receptors. LBD, Ligand binding domain; DBD, DNA binding domain; AF-1, activator function-1; AF-2, activator function-2; NTD, N-terminal domain.

Most nuclear receptors share the same domain structure. Some of these domains are highly conserved; such as the DNA binding domain (DBD) that contains a zinc-finger motif and mediates the interaction of the protein to the DNA, and the ligand binding domain (LBD) that binds the receptor specific agonist or antagonist molecules. DBD and LBD are connected through the hinge in the middle region of the receptor that provides a structural flexibility. In addition, the N-terminal domain (NTD) contains the activator function-1 (AF-1) (ligand-independent) motif, while the LBD contains the activator function-2 (AF-2) (ligand-dependent) motif (6–8) (Figure 1). The hydrophobic pocket within LBD is arranged in a way that gives the pocket a unique character to accommodate specifically the cognate ligands. However, some nuclear receptors have a flexible pocket that binds structurally diverse ligands in various orientations, while others can adopt an active state even in the absence of the ligands (9, 10).

These domains can also be targeted by various post-translational modifications that modulate the nuclear receptor‘s function (11). The binding of the ligand induces conformational changes of the receptor, leading to the recruitment of a complex network of relevant regulatory proteins to complete gene expression activation or inhibition (8). As nuclear receptors can modulate chromatin accessibility, they have the ability to alter the binding of the transcription machinery (6). Nuclear receptors may function as monomers, form a complex of homodimers, or heterodimerize with other nuclear receptors.

**Figure 2:**
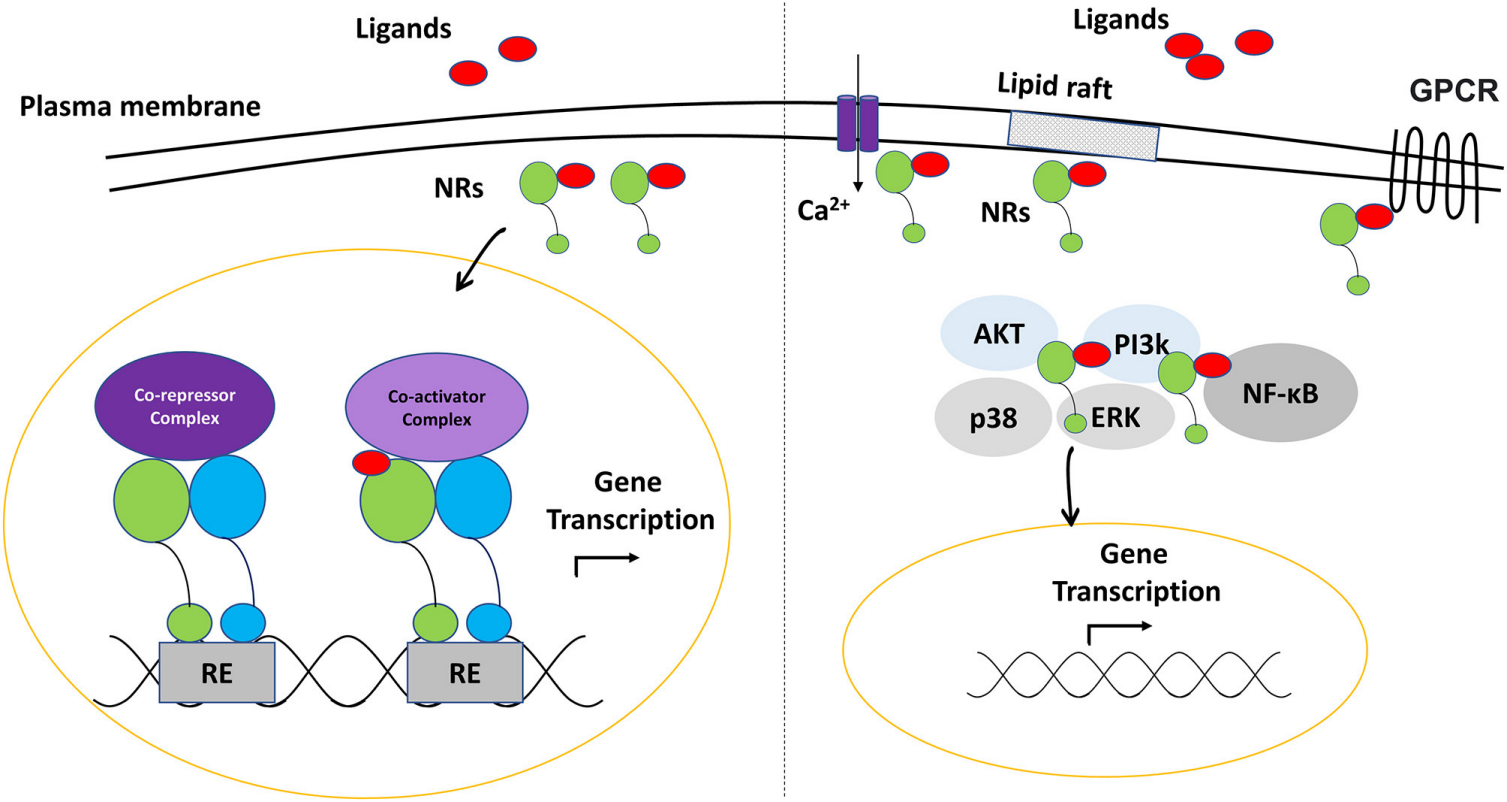
Schematic representation of genomic and non-genomic actions of nuclear receptors.

The most common receptors which act as a heterodimerization partner with other nuclear receptors are the retinoid X receptors (RXRs) (12, 13) (Figure 2). Depending on the dimerization partner, RXR heterodimers are categorized as permissive and non-permissive dimers. In permissive heterodimers (e.g., PPAR/RXR and LXR/RXR), ligand binding to either partner can mediate their activities. While in non-permissive heterodimers (e.g., VDR/RXR and RAR/RXR), the heterodimer remains silent if only RXR is bound, hence, ligand binding to the partners of RXR is a prerequisite for activation (14, 15). While permissive heterodimers seem to be lipid sensors that are regulated by many metabolic pathways (PPAR, LXR), non-permissive ones most likely respond to the classical endocrine steroid (ER) and non-steroid (RAR) factors (16). The crosstalk between nuclear receptors through typical (with RXR as a partner) and atypical (with other nuclear receptors) heterodimerization, as well as the alternative splicing of these receptors, poses a challenge to understand nuclear receptors-mediated gene regulation, and explains the variability in drug responses (17, 18).

### Inflammasomes

Innate immune functions depend on pattern recognition receptors (PRRs) that recognize pathogen-associated molecular patterns (PAMPs), damage-associated molecular patterns (DAMPs) or homeostasis-altering molecular processes (HAMPs) (19, 20). Based on their localization, PRRs are classified to membrane-bound receptors [such as Toll-like receptors (TLRs), C-type lectin receptors (CLR)] and cytoplasmic receptors [nucleotide-binding domain leucine-rich repeat receptors (NLRs), retinoic acid-inducible gene-I (RIG-I)-like receptors (RLRs), absent in melanoma (AIM)-like receptors (ALRs) and proteins-containing tripartite motif (TRIM)] (20–22).

Among the cytoplasmic PRRs, NLRP1, NLRP3, NLRC4, AIM2, and Pyrin have been thoroughly studied and identified as initiators of inflammasome multiprotein complex formation. Although other NLRs; such as NLRP6, NLRP7, NLRP9, and NLRP12 have also been shown capable to form inflammasomes, they need further characterization in order to better understand the conditions of their activation (22, 23).

Generally, NLRs are organized in a tripartite structure: the N-terminal effector domain (pyrin (PYD) for NLRPs; caspase-recruitment domain (CARD) for NLRCs) required for signal transduction, the central NACHT domain (contains NBD) mediates self-oligomerization, and the C-terminal leucine-rich repeats (LRRs) involved in ligand detection (24, 25). In most cases. activation of the NLR triggers a rapid oligomerization process, leading to the recruitment and binding of the inactive pro-caspase-1 enzyme, either directly (NLRC4 inflammasome) or via the ASC adaptor protein (NLRP3 inflammasomes) (22, 26). Eventually, the autocatalytic activation of caspase-1 leads to the processing of GSDMD, pro-IL-1β and pro-IL-18, to induce pore formation and facilitate inflammation or pyroptosis (27, 28).

Depending on the sensor protein of the inflammasome, different types of inflammasomes are activated by different stimuli, for example, NLRC4 recognizes flagellin, NLRP1 recognizes anthrax lethal toxin, and AIM2 is activated by dsDNA (29). However, the triggering stimuli for several inflammasome forming NLRs (such as NLRP2, NLRP6) have not yet been identified. Importantly, while NLRP3 inflammasome is probably the most studied and best characterized inflammasome complex, the direct activator of NLRP3 is still unknown.

### NLRP3 Inflammasome

The assembly of NLRP3 inflammasome mostly requires two events; priming and activation. The priming signal of NLRP3 inflammasome is mostly initiated by TLRs and triggers transcriptional induction of NLRP3 and IL1-β genes via various signaling pathways including NF-κB and MAPKs (JNK, p38, and ERK). Beside transcriptional regulation, priming-induced signaling is also involved in post-translational modifications (phosphorylation, SUMOylation, ubiquitination) that license the inflammasome components (NLRP3 and ASC) for activation (30–32).

The activation step of NLRP3 inflammasome facilitates NEK7 binding, and triggers the assembly of the multiprotein complex. Highly developed modern technologies such as cryo-EM, enabled a detailed structural and mechanistic understanding of NLRP3 inflammasome activation and signaling. It was proven that both PYD and CARD domains are able to form filaments through homotypic interaction. In the absence of a ligand, NLRs are characterized by an autoinhibited conformation, as the LRR folds back to the NBD, resulting in a closed conformation. Sensing of an activator induces a conformational change that leads to the oligomerization of the NLRP3, in a self-propagation process. Thereafter, it recruits ASC through PYD-PYD interaction, and induces the helical ASC filament formation that assembles into large ASC specks, acting as a platform for pro-caspase-1 binding (33, 34).

As a result of intensive studies, a wide range of stimuli and stress signals have been identified for NLRP3 inflammasome activation, many of them are associated with the disturbance of cellular homeostasis or organelle dysfunction. For example, ion fluxes including K^+^ or Cl^−^ efflux, Na2+ influx or elevation of cytosolic Ca^2+^ are required to promote NLRP3 inflammasome assembly and formation (35). Crystals-induced lysosomal destabilization (following engulfment of cholesterol, MSU, β-amyloid) that leads to the cytosolic release of cathepsins and Ca^2+^ results in NLRP3 inflammasome activation (36, 37). Also ROS production following ER stress due to the accumulation of unfolded proteins (38); changes in metabolites and enzymes related to mitochondria function (succinate, itaconate, ATP) (39) or mitochondrial dysfunction (mtROS, oxidized mtDNA, cardiolipin) are proved to be inducers of NLRP3 activation (40). Importantly, mitochondria-associated membranes (MAMs) serve as platform for the NLRP3 inflammasome assembly (36, 41, 42). Indeed, dysregulation of the NLRP3 inflammasome has been linked, and associated with severe diseases, including autoimmune, cardiovascular, neurodegenerative diseases, as well as allergy and cancer, conditions which required careful therapeutic intervention to downregulate the inflammasome network (43–45).

### Nuclear Receptors at the Crossroad of Metabolism and Inflammatory Responses

A growing body of evidence show that nuclear receptors may intervene in responses through various mechanisms. Depending on the microenvironment, immune cells may undergo a wide range of polarization states via transcriptional (re)programming, which in part is regulated by nuclear receptors. The role of several nuclear receptors in the polarization and function of myeloid linage cells has been studied extensity (46, 47). For example, PPARγ or LXR activity is assumed as a hallmark of alternative or M2 macrophage polarization (46, 48). Conversely, under inflammatory conditions, macrophages tend to downregulate PPARγ and its target genes, which diminish the lipid sensing function of these cells (49). However, PPARγ is required during the resolution phase of inflammatory response, and loss of PPARγ is associated with sustained immune response (50, 51).

For proper effector responses, immune cells adopt specific metabolic pathways, where nuclear receptors emerge as key links to fine-tune the immunometabolic effector functions of these cells. Indeed, several nuclear receptors, including FXR, PPARs, LXRs, or PXR, are grouped as metabolic receptors, as they coordinate a network of different metabolic pathways to generally maintain the systemic metabolic homeostasis (3, 52). Recently, active attempts were made to target immunometabolism as an approach to tackle inflammatory disorders (53), and nuclear receptors may function as potential therapeutic modulators at the crossroad of this approach.

Importantly, activation of the NLRP3 inflammasome in THP-1 macrophage was associated with up-regulation of nuclear receptors, suggesting a negative feedback loop promoting resolution of inflammation (54). Consistently, a data mining study showed a strong association between inflammasome and nuclear receptors function, and that inflammasome pathways play a central role in the regulation and expression of nuclear receptors. This study categorized most of the nuclear receptors as sensors or receptors of homeostasis-altering molecular processes (HAMPs). It is a recently introduced expression that refers to alterations in cellular mechanisms and pathways that reflects cellular dysfunction and the loss of cellular homeostasis; consequently, HAMPs may modulate inflammatory responses and associate with several metabolic and immune dysfunction (55).

In response to stress signals, cells initiate a multifaceted program that integrates several highly regulated pivotal processes; including autophagy and inflammatory response (56). Autophagy is a crucial intracellular recycling process that is activated by various types of cellular stress, including protein aggregates, nutrient deprivation, hypoxia, damaged organelles and intracellular pathogens, in order to maintain cellular homeostasis and provide energy source or building molecule blocks for the cell (57). Beside macrophage polarization, several members of nuclear receptors are involved in autophagy-mediated defense at both transcriptional, and post-translational levels, [reviewed in (58)]. In addition, it has been reported that PPARα and FXR regulate autophagy through a complementary transcription of autophagy genes under various nutrition status (59).

Autophagy and inflammasome; as a major inflammatory pathway, are tightly coupled events. Autophagy negatively regulates inflammasome function through several mechanisms, including the removal of endogenous inflammasome activators (DAMPs) or the inflammasome components. However, depending on the conditions, activation of the inflammasome and caspase-1 were found to modulate autophagy, either negatively or positively by various mechanisms (60, 61). Although, nuclear receptor function is associated with autophagy regulation, further experimental data are needed to elucidate the role of nuclear receptors in the crosstalk between autophagy and inflammasome function.

In addition to their role in the transcriptional regulation, nuclear receptors also possess non-genomic activity through the modulation of diverse basic cellular mechanisms, including G-protein signaling, calcium flux, cyclic nucleotide (cAMP and cGMP) or nitric oxide production, and other signal transductions such as a variety of kinase pathways (4). All of which were described to mediate a variety of inflammatory responses, including inflammasome activation/function. Other factors; such as localization of the nuclear receptors in various cellular organelles, increase their potential to develop selective non-genomic activities characteristic to specialized cell types and tissues (4, 62). For example, several nuclear receptors (ER, TR RXRα, RAR, Nur77/TR3 and PPARβ and -γ) were reported to localize in the mitochondria, and become engaged with the mitochondrial transcription, this way, they coordinate the expression of genes encoding for enzymes involved in oxidative phosphorylation (OXPHOS) (63). Importantly, enzymes and products of mitochondrial TCA cycle or OXPHOS have already been recognized as important modulators of NLRP3 inflammasome function.

### Nuclear Receptors and NLRP3 Inflammasomes

Recent studies categorized many of the nuclear receptors as sensors of cellular imbalance and dysfunction. Similarly, NLRP3 inflammasome may be activated by a large spectrum of molecules that are associated with cellular stress and altered metabolism. Data suggest that nuclear receptors are involved in a wide spectrum of cellular processes, and they can regulate inflammasome priming or activation through genomic and non-genomic effects.

Indeed, NLRP3 inflammasome activation may be triggered by lipid metabolites, that are also recognized as ligands by various NRs. For example, the cholesterol and its derivatives [oxysterol (25HC)] are natural ligand for LXR and FXR (64); various fatty acids including saturated fatty acids [palmitic acid (C15:0), stearic acid (C18:0)] (65, 66), mono-unsaturated fatty acids [oleic acid (C18:1)], or polyunsaturated fatty acid [linoleic acid (C18:2)] (67), omega-3 PUFAs [like eicosapentaenoic acid (EPA) and docosahexaenoic acid (DHA)] (68) are recognized by PPARs. Furthermore, enzymes and transporters of lipid metabolism pathways (including synthesis, degradation, or oxidation) that are regulated by various NRs, have also been associated to NLRP3 inflammasome activity as positive or negative regulators (69, 70). Such as fatty acid synthase (FASN) expression is regulated by LXRα (71); while fatty acid oxidation enzymes, NOX4 and carnitine palmitoyltransferase 1A (CPT1A), by PPARγ (72, 73) and PPARα (74), respectively. Expression of CD36 scavenger receptor, that transports cholesterol containing oxLDL to MFs, are regulated by PPARγ (75), while that of the ABCA1/ABCG1, that mediates cholesterol efflux, is regulated by LXR (76, 77). The detailed mechanism of their actions on NLRP3 inflammasome has recently been described in excellent reviews (39, 78–80). In the following section, we highlight known regulatory roles and mechanisms that nuclear receptors exert on NLRP3 inflammasome (Table 1).

**Table 1:** Summary of nuclear receptors and their ligands involved in the NLRP3 inflammasome regulation (arranged in alphabetical order).

**NR**	**Mechanism**	**Effect**	**Cell type**	**Possible therapeutic**	**Used ligand**	**References**
FXR (*NR1H4*)	Interacts with NLRP3 inflammasome components and prevents their assembly, in ligand dependent manner	↓ NLRP3 inflammasome	Peritoneal macrophage, Raw246.7 Sepsis-associated cholestasis mouse model, FXR KO, FXR overexpression in THP-1	Protects mice from septic shock under cholestasis condition	GW-4064, DCA, CDCA, OCA	(81)
FXR (*NR1H4*)	Inhibits ER stress, attenuation of CHOP-dependent NLRP3 overexpression and inhibition of PERK activation	↓ NLRP3 inflammasome	Mouse primary hepatocytes, AML12 cell line, FXR KO mouse	Inhibits ER stress, ameliorates hepatocyte death and liver injury	GW4064, chenodeoxycholic acid (CDCA)	(82)
FXR (*NR1H4*)	Inhibits oligomerization and ubiquitination of ASC	↓ NLRP3 inflammasome	BMDM, peritoneal macrophages, PBMCs (FXR KO mice)	Ameliorates partially the symptoms of NLRP3-related disease models	GW4064, INT747	(83)
LXR (*NR1H*)	Induces the expression of IL-1β, caspase-1, and NLRP3; Metabolic rewiring through Acetyl-CoA accumulation	↑ NLRP3 inflammasome	Human monocytes	Induction of trained innate immunity	GW3965, T1317 (agonist), GSK2033 antagonist)	(84)
LXRβ (*NR1H2*)	Activates P2X7 receptor (non-genomic activity)	↑ NLRP3 inflammasome	Colon cancer cell lines	As a target for Caspase-1 induced pyroptosis	T0901317 (agonist), LXRβ siRNA	(85)
PXR (*NR1I2*)	Enhances the expression of NLRP3 through binding to its promoter region	↑ NLRP3 inflammasome	HUVECs, HepG2	Protects against xenobiotic agents	Rifampicin, SR12813 (agonists) sulforaphane (antagonist)	(86)
PXR	Triggers release of ATP through pannexin-1 channels, P2X7 activation	↑ NLRP3 inflammasome	Human macrophages (THP-1), Mouse Peritoneal macrophages, Nlrp3 KO and PXR KO mice	Link xenobiotic exposure to the innate immunity as potential conserved mechanism	Rifaximin and SR12813 (for human); 16a-carbonitrile (PCN) (for mouse)	(87)
PXR (*NR1I2*)	Blocks NF-κB binding to the promoter of NLRP3	↓ NLRP3 inflammasome	HUVECs	Statin via FXR act as an anti-inflammatory agent	Rifampicin, SR12813, mevastatin, simvastatin	(88)
PPARα (*PPARA, NR1C1*)	Knock out of PPARα associated with up regulation in the inflammasome components and hyper activation of NF-κB pathway	↓ NLRP3 inflammasome	*In vivo*, PPARα KO mice subjected to P. aeruginosa, lung tissue	Protects lung tissue upon lung bacterial infection	Not used	(89)
PPARγ (*PPARG, NR1C3*)	Downregulates the expression inflammasome components	↓ NLRP3 inflammasome	Radiation-induced acute intestinal injury rat model, peritoneal macrophage	Reduces radiation-induced intestinal inflammation	Rosiglitazone	(90)
PPARγ (*PPARG, NR1C3*)	Upregulates IFN-β which required for suppression of NLRP3-mediated IL-1β	PPARγ KO mice exhibit downregulation of NLRP3 inflammasome activation	PPARγ KO mice, Peritoneal macrophages, *in vivo* peritoneal cytokine assessment	Anti-inflammatory	T0070907 (antagonist) Rosiglitazone	(91)
PPARγ (*PPARG, NR1C3*)	Inhibition of IL-1β, caspase-1 and NLRP3 expression	↓ NLRP3 inflammasome	*In vivo*, spinal cord injury (rat). Isolated Neurons from the spinal cord after spinal cord injury (SCI)	Anti-inflammatory protective effects in spinal cord injury	Rosiglitazone, G3335 (antagonist)	(92, 93)
PPARγ (*PPARG, NR1C3*)	Direct binding of PPARγ to NLRP3, inhibits its assembly	↓ NLRP3 inflammasome	Peritoneal macrophages, PPARG^C/−^ mice, (HEK293T cells/system), PBMCs	Regulate metabolic DAMPs- induced NLRP3 inflammasome	Rosiglitazone	(94)
PPARδ (*PPARD, NR1C2*)	Modulation of AMPK activity and ROS production, downregulates IL-1β, caspase-1 and NLRP3, NLRP6, NLRP10	↓ NLRP3 inflammasome	NAFLD mouse model, HepG2	Ameliorates non-alcoholic fatty liver disease associated inflammation	GW501516	(95)
PPARß/δ (*PPARD, NR1C2*)	Downregulation the expression of NLRP3 inflammasome components	↓ NLRP3 inflammasome	MPTP mouse model of PD	Suppress NLRP3-mediated neuroinflammation in PD mouse model	GW501516	(96)
RAR (*RAR*)	Enhances LPS -induced priming signal and glycolytic activity	↑ NLRP3 inflammasome	Human macrophage- derived monocyte	Enhances inflammatory responses	ATRA	(97)
RAR (*RAR*)	Reduced level of ROS and endotoxins in circulation	↓ NLRP3 inflammasome	Rat, *in vivo* study, total blood	Ameliorates alcohol toxicity in the brain	ATRA	(98)
RAR, RXR (*RAR*) (*RXR*)	Enhances flagellin-induced NF-κB/AP-1 activity, and TLR co-receptor CD14	↑ IL-1β Release	THP-1 (human monocyte)	Enhances flagellin-induced proinflammatory responses	ATRA, BMS753 (RARα agonist), LG100268 (RXRα agonist)	(99)
REV-ERBα (*NR1D1*)	Suppress NLRP3 transcription by binding to its promoter, and inhibit NF-κB signaling	↓ NLRP3 inflammasome	REV-ERBα KO mice Peritoneal macrophage, RAW264.7, DSS-induced colitis mouse model	Suppress experimental colitis	SR9009, hemin, GSK4112	(100)
REV-ERBα (*NR1D1*)	Repress NLRP3 and IL-1β transcription by binding to its promoter	↓ NLRP3 inflammasome	REV-ERBα KO mice RAW264.7, human macrophage, peritonitis and LPS/D-Galactosamine-induced fulminant hepatitis mice models	Suppress experimental peritonitis and fulminant hepatitis	SR9009, hemin, SR10067 (REV-ERBα/β agonist)	(101)
RORγ (*RORC, NR1F3*)	Response elements for RORγ in the promoters of the Nlrp3 and Il1b genes	RORγ KO mice exhibit downregulation of NLRP3 inflammasome	BMDMs (RORγ KO mice), human primary macrophages, RAW267	Anti-inflammatory	SR2211, SR1555 (inverse agonist)	(102)
SHP (*NR0B2*)	Inhibits interaction between NLRP3 and ASC	↓ NLRP3 inflammasome	BMDM, SHP KO mice, HEK293T, THP-1	Anti-inflammatory, protect against excessive pathologic responses, preserve the mitochondrial homeostasis	Fenofibrate, AICAR	(103)
VDR (*VDR, NR1I1*)	Attenuates hyperosmotic stress-induced oxidative stress through activation NRF2-antioxidant axis	↓ NLRP3 inflammasome	Immortalized primary human corneal epithelial cell	Potential therapeutic agent to treat dry eye problems	Calcitriol (1,25-D)	(104)
VDR (*VDR, NR1I1*)	Suppress NF-κB nuclear translocation through binding to importin 4	↓ NLRP3 inflammasome	MRL/lpr mice, Mouse renal tubular epithelial cells	Potential therapeutic agent for lupus nephritis	Paricalcitol (agonist)	(105)
VDR (*VDR, NR1I1*)	Inhibits NLRB3 binding to NEK7, NLRP3-mediated (ASC) oligomerization, and (ROS) accumulation	↓ NLRP3 inflammasome	Peritoneal macrophages, DSS-induced ulcerative colitis mouse model	Attenuates DSS-induced acute colitis in mice, inhibits CD4+ T cells generation and polarization Decrease M1/M2 macrophages ratio	Calcitriol (1,25-D)	(106)
VDR (*VDR, NR1I1*)	Enhances IL-1β secretion mediated by NLRP3 inflammasome	↑ NLRP3 inflammasome	PMA-differentiated THP-1	Enhances inflammatory response	1,25(OH)2D3, 25(OH)D3, 7DHC, vitamin D3	(107)
VDR (*VDR, NR1I1*)	Direct interaction with NLRP3, Inhibits BRCC3-mediates NLRP3 deubiquitylation	↓ NLRP3 inflammasome	BMDMs, mouse peritoneal macrophages (Vdr KO mice)	Anti-inflammatory, increases the survival rate in murine models of sepsis	1,25-D	(108)
VDR (*VDR, NR1I1*)	Modulates AhR/NF-κB signaling, downregulate inflammasome components	↓ NLRP3 inflammasome	Periodontitis mouse model, gingival epithelium	Alleviates the periodontitis	1,25-D	(109)

*If it is not stated as other, then listed ligands are agonists*.

#### Retinoid X Receptor (RXR)

RXRs are involved in the transcriptional programs of many biological processes, including cell differentiation, immune response, as well as lipid- and glucose metabolism. The importance of RXRs; as master nuclear receptors, are derived from their ability to play a unique role as obligate heterodimerizing partners of many other nuclear receptors (110). In fact, there is different affinity and competition between RXR partners to form heterodimers with RXR (111). In addition to its role as transcription factor in the nucleus, activation of RXR may induce its translocation into the mitochondria, in order to enhance the transcription of mitochondrial genes (such as COX-1) (112).

In mouse and human myeloid cells, RXRs have multifaceted functions, including polarization, uptake of apoptotic cells, inflammatory gene repression, cholesterol uptake and lipid processing [extensively reviewed in (113)]. For example, RXR is linked to IL-4-induced alternative polarization of macrophages, via modulating the phenotypic properties of this macrophage in gene-specific manner (114). During the years, many RXR-specific synthetic ligands and RXR-deficient mice were generated to avoid heterodimerization issues; which is regarded as a drawback in RXR-targeted therapeutic approaches. Currently, it seems that partial agonists for RXR with submaximal response could be an approach to avoid prolonged activation and side-effect (115).

**Figure 3:**
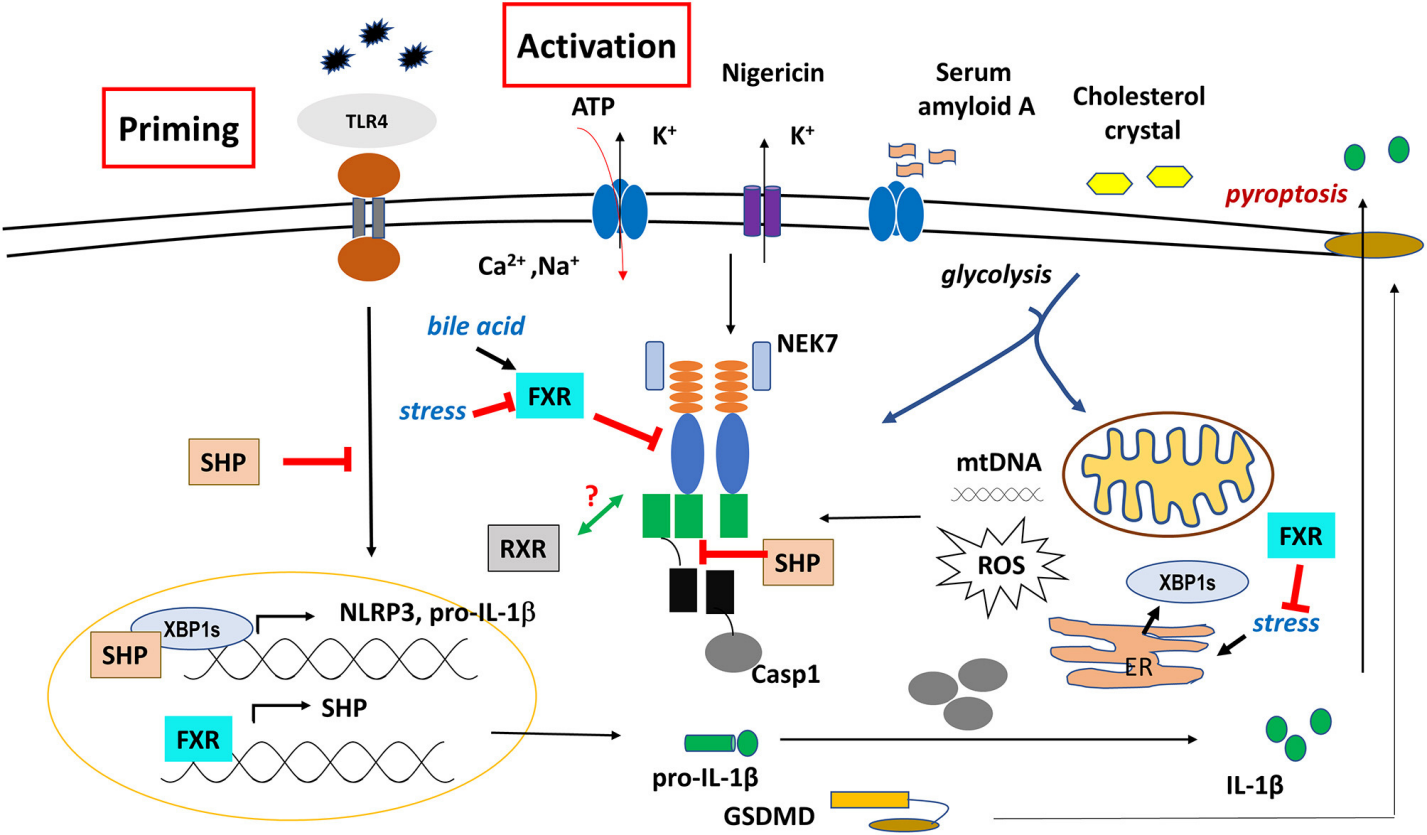
Regulatory role of SHP, FXR, and RXR on NLRP3 inflammasome functions. The effect of SHP is controversial, probably it depends on the cell or tissue type.

Unfortunately, little is known about the exact role of RXRs in inflammasome regulation, as most studies focus on their dominant partners, particularly in permissive dimers. Interestingly however, one report suggested that NLRP3 inflammasome activation may regulate RXRs function (Figure 3). In ovalbumin-induced mice model of eosinophilic asthma, NLRP3 inflammasome activation was required for the induction of allergic response (116). They showed that NLRP3 specific inhibitor MCC950 significantly reduced both RXR expression and apoptosis in primary airway epithelial cells (pAECs), however, inhibition of apoptosis was reversed by the RXR agonist adapalene. Although an NLRP3-RXR axis was suggested as important regulatory role for apoptosis, further studies are required to delineate the mechanism, and find direct association between NLRP3 and RXR.

#### Small Heterodimer Partner (SHP)

The orphan SHP is a unique nuclear receptor that lacks DBD. Though it has a classical LBD that contains a ligand-dependent transactivation domain (AF2), there are two NR-boxes within its NTD that mediate binding of other proteins. Interestingly, the SHP interacting proteins are mainly nuclear receptors (such as FXR, ER, LXR, PXR, RAR, CAR, and PPAR) or other transcription factors (such as BMAL1, c-Jun, SREBP-1c, FOXO1, p65, and USF1), and dimerization with SHP usually leads to transcriptional repression of their target gene (117). These interactions regulate complex networks of metabolism, hemostasis and immune responses (52, 117, 118).

While the liver expresses SHP at a highest level, it is also expressed in various other tissues (such as, heart, adipose and intestinal tissues) in both human and mouse (119). In human, a number of mutations and single nucleotide polymorphism (SNP) were found to affect the function of SHP (117). The expression of SHP is transcriptionally regulated by ligated FXR, in order to regulate the biosynthesis of bile acids and cholesterol (52). SHP was shown to modulate TLR activation through interaction with downstream signaling NFκB and TRAF6, resulting in the attenuation of proinflammatory response (120).

In BMDMs, loss of SHP was shown to be associated with excessive pathologic responses, mediated by NLRP3 inflammasome activation. Though SHP did not affect the priming signal of NLRP3 inflammasome, it had the ability to compete with ASC for NLRP3 binding following treatment with various NLRP activators (ATP, MSU, nigericin), hence, it negatively regulated the activation and assembly of NLRP3 inflammasome (Figure 3). In addition, SHP directly associated with NLRP3, ASC, TXNIP and MAVS on mitochondrial membrane, in order to help mitochondrial translocation of NLRP3 inflammasome as well as to regulate mitochondrial homeostasis (103).

In pancreatic acinar cells however, thapsigargin treatment upregulated SHP expression that in turn, stabilized the spliced isoform of X-box-binding protein 1 (XBP1s) transcription factor, a key mediator of ER stress response (121). As ER and ER stress play a critical role in inflammasome activation by several mechanisms (such as ROS production) (43), this observation would predict the role of SHP as a facilitator of inflammasome function. In notion with this hypothesis, it was recently reported that XBP1s bound to the promoter region of NLRP3, and loss of XBP1 in renal ischemia/reperfusion- mediated injury was associated with a decrease in NLRP3-mediated caspase-1 activation (122). It seems that XBP1s' function is more likely to be tissue-specific, and depends on the testing conditions. In this context, SHP could be a potential target to modulate inflammasome function either directly, or via the regulation of XPB1s.

#### Farnesoid X Receptor (FXR)

FXR; as a transcription factor, binds to DNA either as a monomer or as a heterodimer with RXR. Together with PXR and VDR, it belongs to the group of metabolic nuclear receptors, and is basically considered as a sensor for endogenous bile acids (BAs), and a key regulator for bile acids homeostasis (123). It is mainly expressed in the liver and intestine, as well as in various immune cells (including macrophages, dendritic cells (DCs) and T-cells) (124, 125).

Studies show that FXR mediates cell-type specific functions. In hepatocytes, FXR, as a key regulator of bile acids synthesis, senses both primary and secondary bile acids with different affinity, and initiates transcriptional regulation of gene sets; including SHP, in order to inhibit further bile acid synthesis, in a negative feedback mechanism. SHP mediates transcriptional repression of several genes; including CYP7A1, a rate-limiting enzyme in bile acid synthesis. The intestinal FXR mediates bile acids transportation through regulation of several transporters involved in this process. Furthermore, upon activation, FXR mediates fibroblast growth factor 15 (FGF-15) (FGF-19 in humans) secretion, which translocates through portal circulation, and binds the basolateral fibroblast growth factor receptor 4 (FGFR4) in hepatocytes, mediating transcriptional inhibition of *CYP7A1* (123, 125, 126). While FGF is important for bile acids, glucose, and lipid homeostasis, protecting against hepatosteatosis, in mice fed a high fat diet, lack of FGF results in increased hepatosteatosis, associated with endoplasmic reticulum (ER) stress and impaired tissue regeneration (127). Furthermore, activation of FXR/SHP axis also inhibits PXR-, LXR-, and PPARα-mediated *CYP7A1* gene transcription, and reduces the expression of SREBP1, a member of the basic helix-loop-helix-leucine zipper (bHLH-Zip) transcription factor family, that is involved in lipogenesis (127–130).

Recent reviews demonstrated that the function of FXR is far more than being simply a bile acids regulator, as it participates in a dynamic network of lipid and glucose metabolism, it maintains the homeostasis in different tissues and organs, and has various regulatory roles in innate immune responses (125, 131). Like in hepatocytes and macrophages FXR inhibits NFkB signaling and suppress proinflammatory cytokine secretion under inflammatory conditions (132, 133). Furthermore, it mediates M2 polarization of macrophages, and modulates DC differentiation to promote anti-inflammatory effects. In mouse colitis model, it upregulates IL-10 expression and reduceses DC and effector T cell infiltration, while recruits Treg to the colonic inflammatory site (123–125).

FXR was originally shown to be activated by an excessive level of farnesol; an intermediate metabolite of the mevalonate pathway. In fact, dysregulation of the mevalonate pathway has been linked to the innate immune function and Pyrin- and NLRP3 inflammasome activation (134, 135). Activation of FXR by various ligands leads to the upregulation of CYP3A expression, which hydroxylates ATRA, an RAR agonist, and accelerates its catabolism. This indicates that FXR may regulate RAR-mediated signaling by modulating ATRA availability (136). Furthermore, FXR is known as a repressor of autophagy, and the activation of FXR induces transcriptional repression of autophagy genes (such as such as, *Ulk1, Lc3a, Lc3b, Gabarap, Wipl1, Wipl2*, and several *Atg* genes) (137), and autophagy protein and vesicles even under nutrient deprivation condition (123).

The effect of FXR on inflammasome-related functions and cytokine secretion seems to be complex, and in some cases controversial, possibly due to differences in the studied conditions. The activation of macrophages and Kupffer cells by bile acids induces the secretion of inflammatory cytokines; such as IL-1β and TNF-α, which in turn, also inhibit *CYP7A1* gene transcription through activation of PKC and JNK kinase signaling pathways (129). Any defect in the flow of bile acids was delineated as cholestasis, and can be associated with sepsis (138). FXR-null mice exhibited deregulation in bile acids, glucose and lipoprotein metabolism, and had a high risk of hepatocellular carcinoma (HCC) and other degenerative liver diseases, in addition to abnormalities in the function of the intestinal-epithelial barrier (139–141). Furthermore, activation of intestinal FXR by bile acids induced the expression of a group of genes involved in mucosal defense, which inhibited bacterial overgrowth and prevented deterioration of the intestinal epithelial barrier (142).

In human and mouse differentiated macrophages, increase in bile acids over the physiological level is considered as a DAMP, and can initiate the priming signal (signal 1) by enhancing the expression of IL-1β and NLRP3, and activate the NLRP3 inflammasome through promoting prolonged calcium influx (signal 2) (81, 138) (Figure 3). In sepsis-associated cholestasis mouse model, FXR-null mice were found to be more susceptible to LPS-induced death, had high level of bile acids in the serum, and the isolated peritoneal macrophages exhibit higher activation of caspase-1/IL-1β compare to the wild type cells under LPS challenge. Treatment with cholestyramine resin; a bile acid sequestrant, or overexpression of FXR, restored the serum level of bile acids, improved their survival, and repressed the caspase-1 and IL-1β activation. However, intraperitoneal treatment with GW4064; an FXR agonist, did not protect the mice from this septic shock. Importantly, a co-immunoprecipitation analysis revealed that FXR may physically interact with NLRP3 inflammasome components, hence, prevent their assembly and repress the activation of NLRP3 inflammasome in a ligand-independent manner (81).

Conversely however, other report showed that several bile acids derivatives suppressed LPS/nigericin-induced NLRP3 inflammasome activation in BMDMs, through TGR5-cAMP-PKA axis, mediating ubiquitination and subsequent phosphorylation of NLRP3 at a single residue (Ser 291), which led to the inhibition of NLRP3 inflammasome activation (143). However, activation of FXR by its agonist INT-747 had no effect on NLRP3 ubiquitination, suggesting that FXR is not involved in bile acid-induced NLRP3 inflammasome suppression (143).

Recently, a study showed that down-regulation of FXR expression correlated with the high expression of inflammasome-associated genes, and induced inflammasome activation in various liver diseases. Furthermore, tunicamycin-induced ER stress animal model exhibited down-regulation of hepatic FXR, in association with up-regulation of NLRP3 and TXNIP levels and NLRP3 inflammasome activation. These effects were exaggerated under the loss of FXR, while treatment with FXR agonist GW4064 ameliorated NLRP3 activation. They also showed that under ER stress, loss of FXR resulted in the up-regulation of p-PERK, and CHOP; typical regulators of ER stress response (82). Notably, during the ER stress response, PERK and XPB1 mediate CHOP expression, which is involved in apoptosis and NLRP3 inflammasome activation, leading to caspase-1 cleavage, IL-1β secretion and pyroptosis. CHOP as a transcriptional regulator mediates NLRP3 and TXNIP expression (82, 144). In line with these results, ligated FXR inhibited p-PERK- and CHOP- mediated ER stress and subsequent NLRP3 inflammasome activation. This inhibition was most likely due to the attenuation of miR-186-mediated inhibition of NCK1, which is a regulator of PERK/CHOP axis (82).

Because of their safety profiles and proven efficacy, several steroid and non-steroid FXR agonists; such as GW4064, WAY362450, Px-104, and INT747, were developed to overcome liver- associated diseases (145). However, they still require further investigation, to understand their impact on the inflammatory mechanisms. In BMDMs, FXR agonist GW4064; but not INT747, inhibited NLRP3 and AIM2 inflammasomes activation in an FXR-independent manner, while it had no effect on NLRC4 inflammasome. This molecule had the ability to attenuate ASC oligomerization through the reduction of ASC ubiquitination, leading to impairment in both NLRP3-ASC interaction and ASC oligomerization (83). It was reported that the expression of FXR was downregulated following hepatic ischemia reperfusion injury (IRI) in mouse model. In this model, deletion of FXR was associated with induction of NLRP3 inflammasome-mediated GSDMD-N and caspase-1 cleavage, and an increased pyroptosis-mediated liver damage was observed (146). In IRI model, GW4064 treatment reduced the secretion of proinflammatory cytokines, including that of IL-1β. Compared to BMDMs, FXR is highly expressed in Kupffer cells. Knock-down of SHP or depletion of Kupffer cells abolished FXR-mediated inhibition of liver inflammatory genes (147).

Altogether, these findings suggest that FXR plays a crucial role in the regulation of inflammasome function. However, the role of FXR is influenced by various factors, including the expression level of FXR in different cells and tissues, the type and differentiation status of the immune cell, study conditions, or the type and affinity of the endogenous/exogenous FXR ligands. Notably, FXR ligands may also have a direct effect on the inflammasome in an FXR-independent manner. Furthermore, bacterial endotoxins markedly downregulate the expression of FXR, which may explain sometimes controversial reports regarding NLRP3 inflammasome. Importantly, FXR may also affect inflammasome function indirectly, by regulating the expression and function of other nuclear receptors (e.g., FXR-SHP axis).

Nowadays, FXR ligands are under clinical trials targeting human metabolic dysregulation-associated diseases; such as obesity, type 2 diabetes, or liver diseases (148). A screening of more than a thousand FDA/EMA-approved drug revealed that some of them modulated the activity FXR, hence, FXR may affect the outcome of various therapies. Nevertheless, FXR may open an opportunity for polypharmacology, particularly in inflammasome-related diseases (148).

#### Pregnane X Receptor (PXR)

PXR exhibits distinctive large, flexible and dynamic LBD to accommodate a broad spectrum of hydrophobic ligands with various structural and physicochemical characteristics. These ligands may include natural steroids (such as progesterone, corticosterone), synthetic drugs (like dexamethasone), antibiotics (like rifampicin), bile acid, herbal compounds and environmental toxicants (149, 150). PXR is considered as a master xenobiotic sensor that; upon activation, heterodimerizes with RXR to regulate a set of downstream genes, such as enzymes of the mitochondrial cytochrome P450 family (mainly CYP3A and CYP2b), various transferases (like GST1) and ABC transporters (like MDR) mainly required in detoxification. Due to its central regulatory role in detoxification, activation of PXR may lead to clinical consequences like drug resistance or drug-drug interactions-induced toxicity that may severely affect the outcome of a therapy (151, 152).

Several molecules and mechanisms have been described as positive, or negative regulators of PXR's activity. Like transcriptional co-activators (SRC-1, RIP140, PGC-1) or co-repressors (NCoR, SMRT) that are recruited to PXR-RXR following ligand binding. Besides, various signaling pathways (NFkB, AP-1, PKA, PKC, CDK, etc.) and post-translational modifications of PXR (phosphorylation, acetylation, SUMOylation, ubiquitination) may potentiate or terminate the activity of PXR. Interestingly, a reciprocal connection between PXR and NFκB pathways has been reported, in which activation of one pathway suppresses the activity, and expression of the target genes for the other (153). Activity of PXR is also regulated via crosstalk with other nuclear receptors (CAR, VDR, HNF4a) or transcription factors (CREB, FOXO, NFkB), like LXR, FXR, or PPARs by sharing or competing for the binding of co-regulatory molecule or response element on the target gene (154–156).

PXR is highly expressed in the liver and intestine, but functions in many other tissues and different cell types, hence, it affects several physiological and immunological functions, including metabolism, inflammation, cell cycle and apoptosis. sometimes with contradictory results. Interestingly, tissue- and species-specific ligands for PXR have also been identified. For example, in the vascular endothelium of the aorta and pulmonary vessels, PXR activation by indole 3-propionic acid (IPA); a circulating tryptophan metabolite of intestinal microbiota, inhibits eNOS-mediated NO production, consequently leading to vasoconstriction of the blood vessels (157). While in mesenteric arteries, vasodilation was induced by the PXR agonist progesterone metabolite, probably via the induction of CYP enzymes. Furthermore, rifaximin which is a gut-specific human PXR ligand, is not effective for rodents; while pregnenolone-16 alpha carbonitrile (PCN) functions as a rodent-specific ligand (158–161).

PXR is also expressed in immune cells; such as including B- and T cells, as well as macrophages. Myeloid-specific PXR deficiency was shown to lead to reduction of atherosclerosis as a result of reduced lipid uptake and foam cell formation (162). PXR ameliorates acute kidney injury by regulating AKR1B7 which attenuates mitochondrial dysfunction, and leads to improvement in lipid metabolism (163). These data suggest that PXR is involved in several mechanisms that are potentially connected to the regulation of NLRP3 inflammasome, which also serves as a sensor for potentially dangerous or foreign molecules.

**Figure 4:**
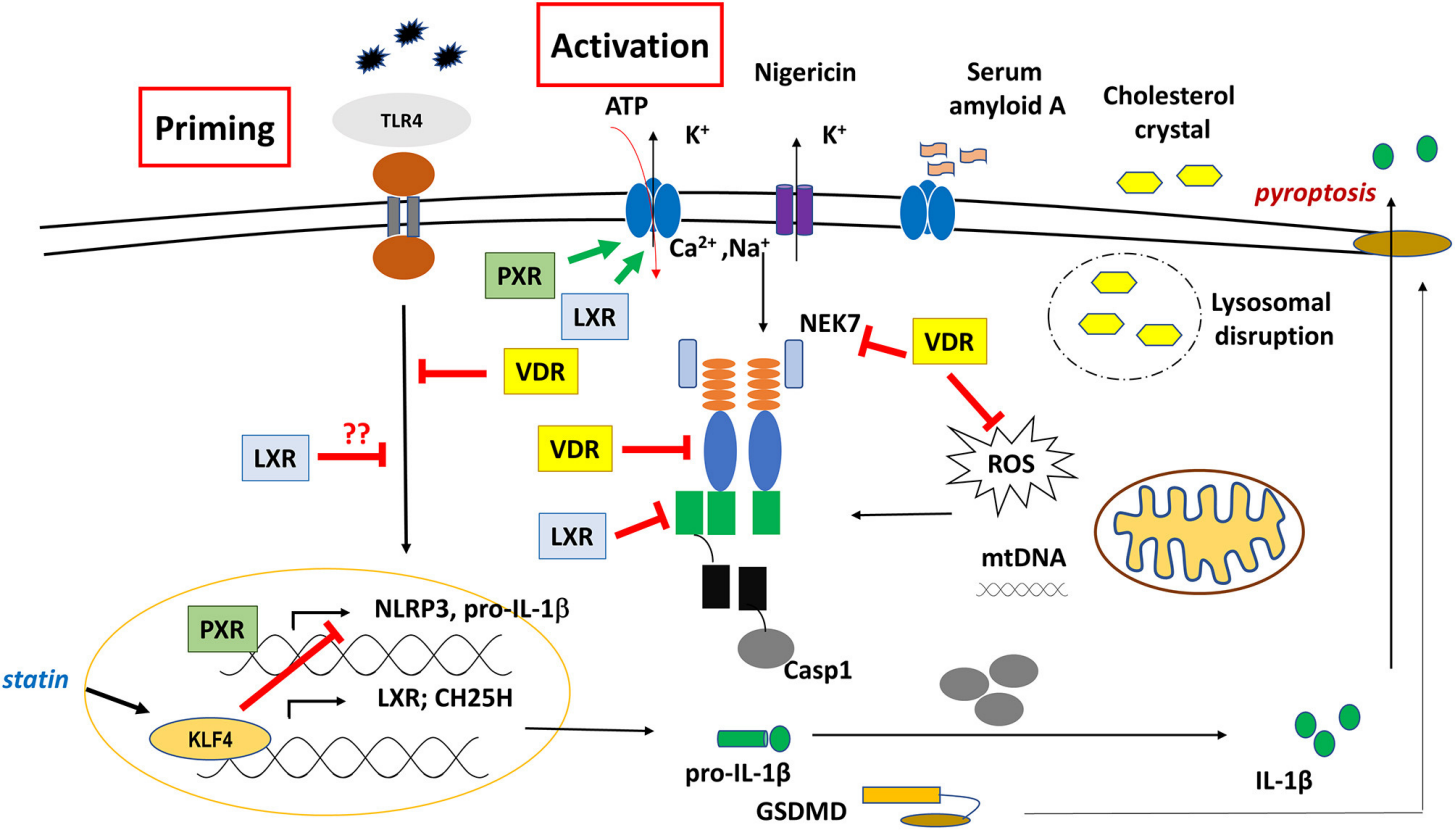
Regulatory role of VDR, PXR, and LXR on NLRP3 inflammasome functions.

It was shown that treatment of human umbilical vein endothelial cells (HUVECs) with PXR agonist (rifampicin or SR12813), or overexpression of PXR induced the expression of various NLRs (NOD1 NLRP1, NLRP3) and TLRs (TLR2, TLR4, TLR9). PXR was shown to induce NLRP3 inflammasome-mediated caspase-1 and IL-1β cleavage, and silencing of PXR or treatment with PXR antagonist (sulforaphane) diminished these effects. Importantly, using CHIP and promoter-reporter based assays, several PXR-responsive elements (PXREs) were identified in the NLRP3 promoter region, indicating a direct regulatory role of PXR in NLRP3 priming signal (86) (Figure 4). In line with this, activation of PXR with species-specific ligands induced NLRP3 inflammasome-dependent caspase-1 activation, and IL-1β release in human THP-1 and LPS-primed mouse macrophage. PXR activation triggered rapid release of cellular ATP through pannexin-1 channel, leading to P2X7 receptor activation and subsequent NLRP3 inflammasome activation, suggesting a non-genomic function for PXR in NLRP3 inflammasome activation (87).

Nevertheless, PXR was reported to mediate the inhibitory effect of statins (simvastatin or mevastatin) on ox-LDL- or TNF-α-induced NLRP3 activation, as well as reduced NF-kB binding to the promoter of NLRP3 in endothelial cells. Knockdown of PXR abolished statin- or PXR agonist-mediated suppression of NLRP3 inflammasome activation (88). Altogether, these data suggest important, yet versatile roles for PXR in innate immunity, and cellular homeostasis under xenobiotic challenges. Considering that both PXR and NLRP3 sense xenobiotics, and a huge variety of (harmful) molecules, they also share and use an overlapping pathway during their function, the two systems deserve further studies in order to delineate cell-specific mechanisms of the potential “crosstalk” between PXR and NLRP3 inflammasome.

#### Liver X Receptor (LXR)

LXRs (LXRα and LXRβ) form permissive heterodimers with RXR. While the expression of LXRα is mainly restricted to adipose tissue, liver, intestine, and macrophages; LXRβ may present in all types of cells and tissues. LXRs are considered as cholesterol sensors, and activation of LXRs with its endogenous agonist (oxysterol) modulates the expression of genes related to lipid metabolism, especially fatty acid synthesis [such as fatty acid synthase (FASN)] and cholesterol efflux (ABCG1) (164). In the liver, LXR senses elevated cholesterol level, and activates the expression of CYP7A1; the rate-limiting enzyme of neutral bile acid synthesis.

At the sites of the atherosclerotic lesions, macrophages usually become foam cells by accumulating excess cholesterol. Importantly, cholesterol crystals in macrophages are important inducers of inflammatory signaling, including inflammasome-mediated IL-1b secretion, which is a major driver of tissue damage developed in atherosclerotic lesions (165, 166). However, in mouse model of atherosclerosis, LXR agonists were shown to dramatically decrease lesion formation (167), through modulating the function of macrophages. LXR agonist induce reverse cholesterol transport (RCT) in macrophages by enhancing the expression of cholesterol efflux transporter ABCA1 and ABCG1 (168). However, LXR not only mediates transactivation of genes with LXR responsive elements for RCT in macrophages. LXR may also attenuate the inflammatory response in a ligand-specific SUMOylation-dependent “transrepression” mechanism. Interestingly, upon binding of synthetic agonist (GW3965) or some endogenous oxysterol, such as 22R, 24(S), 25EC, and 24HC; but not 25HC or 27HC, LXR is targeted to sumo-modification to recruit co-repressor NCoR/SMART molecules. The whole complex then interacts with NFκB, and is recruited to NFκB binding site of proinflammatory target genes, in order to inhibit their expression (169, 170). Thus, it seems that while intracellular sterols trigger inflammatory responses, at the same time, they also activate LXR, which in turn initiates an anti-inflammatory pathway to limit inflammation (171). As the effect of LXR on inflammatory responses is still controversial due to the various model systems (172, 173), the potentially regulatory role of LXR on the NLRP3-mediated mechanisms also requires further in-depth studies.

Beside modulating NFkB signaling, LXR possess other forms of non-genomic functions to regulate NLRP3 inflammasome functions. LXRs were found to be expressed in various cancer cell lines, and regulate cell proliferation, and cell death. In both human and mouse colon cancer cell lines, it was shown that T0901317; a synthetic ligand for LXR, induced NLRP3 inflammasome-mediated caspase-1 activation. Mechanistically, it was shown that the ligand-activated LRXβ interacted with pannexin-1. Activation of the channel resulted in ATP secretion, and eventually, it led to the activation of P2X7 purinergic receptor; a major driver of NLRP3 inflammasome activation. The ability of LXRβ to mediate caspase-1 activation was associated with pyroptosis-mediated cell death that resulted in the reduction of tumor growth in mice model (85) (Figure 4). This mechanism may provide more insight about LXR-β/pyroptosis axis as a target candidate for cancer therapy.

Cerebral inflammatory demyelination is the major feature of childhood cerebral ALD (CCALD). In an iPSC model of CCALD using microarray-based transcriptional profiling analysis, significant up-regulation of cholesterol 25-hydroxylase (CH25H) expression was detected, and in line with this, an elevated 25-hydroxycholesterol (25-HC) (typical agonist of LXR) was measured, which are major risk factors of cerebral inflammation and contributors of neurodegenerative diseases (174). Following *in vivo* injection of 25-HC into the corpus callosum of wild-type mouse brains, recruitment of Iba1 positive microglia and elevated level of IL-1β was detected in the injected region. *In vitro* treatment of LPS-activated BMDM with 25-HC also increased IL-1β secretion via NLRP3 inflammasome activation. They showed that 25-HC enhanced K-efflux and the production of mtROS, and these mechanisms could be inhibited by the LXR antagonist, 22(S)-hydroxycholesterol [22(S)-HC] (175).

However, importantly, a later study identified Krüppel-like factor 4 (KLF4) as an important inducer of CH25H expression in endothelial cells as well as in macrophages (176). KLF4 is a master transcription factor that regulates anti-inflammatory responses, aiding in the transition of macrophages from M1 to M2 phenotype (176, 177). This study showed that the promoter region of LXRα possessed binding sites for KLF4, and that the overexpression of KLF4 induced the expression of LXRα and cholesterol 25-hydroxylase (CH25H), while it inhibited the expression of the NLRP3 inflammasome components. Furthermore, using LC-TMS, they detected an elevated concentration of 25-HC in KLF4-transfected THP-1 monocytes. Treatment of BMDM with 25-HC resulted in increased CH25H and LXR; and decreased NLRP3 levels. The study concluded that atheroprotective factors (such as statins or pulsatile shear stress) induced KLF4 expression in endothelial cells and macrophages, which mediated expression of CH25H and LXR. Due to the activity of CH25H, intracellular 25-HC level was elevated; which acting as an LXR agonist, induced cholesterol efflux genes (like ABCA1) and suppressed pro-inflammatory gene expression, including that of the NLRP3 inflammasome components (178).

Similar to these findings, using LPS-primed BMDM or peritoneal macrophages, synthetic LXR agonists (T0901317; T09) significantly inhibited NLRP3 inflammasome activation by abolishing ASC oligomerization, mtROS generation, and the transcription of NLRP3 and pro-IL-1β (179). In line with these results, LXR synthetic agonists T09 significantly downregulated the expression of NLRP3 and caspase-1 in microglia that were activated with intravitreal injection of Amyloid β (Aβ1-40) (178). The inhibitory effect of T09 was explained by the inhibition of IkB phosphorylation.

Another study aimed to delineate the potential protective role of LXR following irradiation, in order to enhance shifting of macrophage polarization from M2 to M1 phenotype, which is a preferred form to fight in tumorous conditions. Using immortalized BMDMs (iBMDM), LXR activation was found to mediate protection against ionizing radiation. Following irradiation in LXR-deficient BMDMs, significantly increased p53 expression, caspase-1 activity and subsequent pyroptosis, as well as pro-inflammatory cytokine production was detected. They concluded that ionizing radiation of LXR-deficient or LXR antagonist–pretreated macrophages promoted macrophage polarization toward a pro-inflammatory (M1) type, however, irradiation markedly reduced their viability as a result of the enhanced pyroptosis (180).

On the contrary however, the activation of LXR by synthetic ligands is associated with induction of the trained innate immunity and proinflammatory phenotype in human monocytes. The trained innate immunity was dependent on NLRP3 inflammasome-mediated IL-1β production, which was associated with up-regulation in the expression of NLRP3 and IL-1β upon LXR activation (84).

LXR has been also reported to regulate IL-18 expression in primed BMDM via multiple mechanisms. It was shown that activation of LXR downregulated the LPS-induced gene expression of IL-18, pro-IL-1β and pro-caspase-1, without effecting NLRP3 expression. However, at the same time, LXR activation induced the expression of IL-18BP; an IL-18 decoy receptor. Mechanistically it was shown that activation of LXR augmented the binding of IRF8 transcription to the promoter of IL-18BP, this way facilitating IL-18BP expression that consequently participated in the reduction of IL-18 cytokine level (181).

These results altogether indicate, that though LXR may regulate NLRP3 inflammasome function at multiple level, further studies are needed to clarify molecular mechanisms.

#### Peroxisome Proliferator-Activated Receptors (PPARs)

PPARs regulate gene transcription by forming permissive heterodimers with retinoid X receptors. They are generally activated by a diverse group of fatty acids and their derivatives, a variety of eicosanoids, and a number of selective synthetic drugs. PPARs coordinate lipid and glucose metabolism, and they are drug targets in hypertriglyceridemia and insulin resistance. Dysfunction of PPARs may lead to perturbation of lipid metabolism that is characteristic in metabolic syndromes (obesity, atherosclerosis), and is accompanied by low-grade chronic inflammation termed “sterile inflammation” with continuous production of pro-inflammatory cytokines including IL-1β. At the same time, dysregulated lipid metabolism may also lead to the decrease of endogenous lipid ligands for PPARs, resulting in the disturbance of mitochondrial development and function, release of mitochondrial DNA, and elevated mtROS. Elevation of the cytosolic danger signals (such as mtDNA and mtROS) develop cellular stress that may eventually lead to enhanced inflammatory responses (182). A thorough review has been published recently about the role of PPARs in immune responses (183). Importantly, though all PPARs play a major regulatory role in energy homeostasis, each of them has a characteristic location of expression and distinct functions in the tissues, hence, they may intervene in inflammatory responses by various way.

##### PPARγ

PPARγ is mostly expressed in white adipocytes, but can also be found in the kidney, liver, intestine, skeletal muscle, breast, prostate as well as in a variety of immune cells like macrophages, dendritic cells, eosinophils, T cells and B cells (184, 185). Increased expression of PPARγ was documented at sites of inflammation such as in arthritis, colitis, and in foam cells from atherosclerotic plaques. PPARγ is essential to the regulation of white adipocyte differentiation, and plays a critical role in lipid (energy) storage, glucose metabolism and insulin sensitivity. In brown adipose tissue, it regulates the expression of mitochondrial proteins (such as the PGC-1 txnal coregulatory of PPARg, or UCP-1 responsible for thermogenic respiration), and members of the electron transport chain. In macrophages, it drives foam cell formation in atherosclerosis, as it induces the expression of CD36 scavenger receptor and fatty acid transporter that regulates oxLDL uptake into the cells (186, 187). PPARγ is activated by naturally occurring ligands, including prostaglandins (15deoxy-PGJ2), and oxidized low density lipoprotein (ox-LDL), as well as by synthetic agents such as thiazolidinedione antidiabetic drugs and non-steroidal anti-inflammatory drugs (NSAIDs), thus, PPARγ is the most common PPAR isoform targeted for therapeutic interventions.

**Figure 5:**
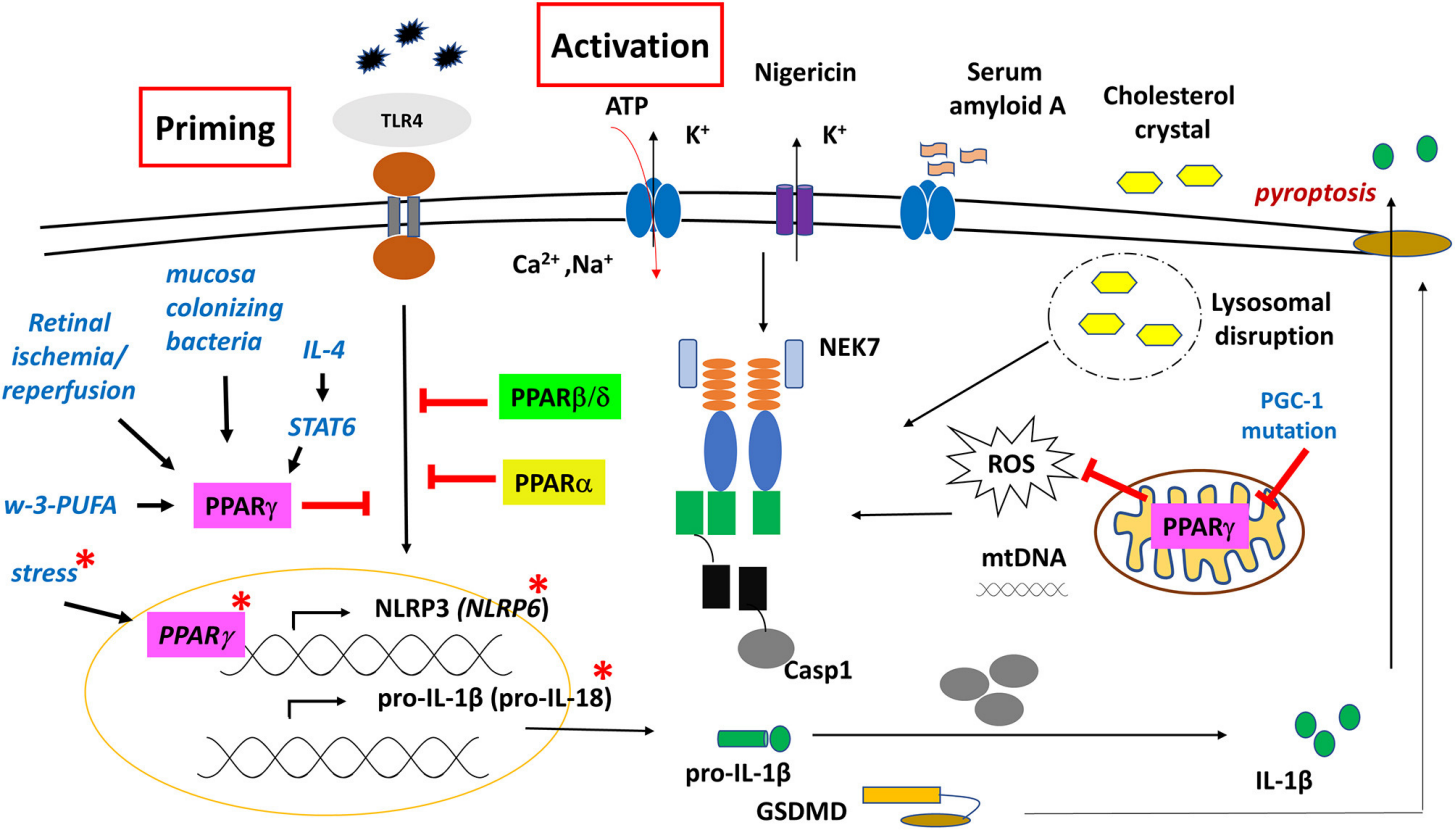
Regulatory role of PPARs on NLRP3 and NLRP6 inflammasome functions. PPARγ has both genomic and non-genomic effects on NLRP3 inflammasome function. Red asterisk indicates molecular events in the gut epithelial cells.

Regarding inflammatory responses, while PPARγ does not regulate MF differentiation, it was shown to exert a variety of anti-inflammatory effects, in part, by repressing numerous NFκB target genes. These effects are either mediated through direct physical interactions and sequestration of NFκB coactivators, or in a DNA-independent way, mainly by impairing phosphorylation of the signaling factors. Early studies have reported that the activation of PPARγ inhibited inflammatory responses, including pro-inflammatory cytokine secretion (188–190). These studies also showed that signaling pathways (p38, NFκB, ERK1/2) activated via TLR2 and TL4 were inhibited by PPARγ in various condition, such as in T2D, cardiomyopathy, or in MSU crystal-induced acute inflammation in gout, aiding in the resolution of the inflammatory response (191–195) (Figure 5). Later, several studies proved that inflammatory responses highly associated with deficiencies in lipid metabolism are mediated through PPARγ.

Regarding inflammasome, an early study using CGI-58 (Comparative Gene Identification-58) deficient mice fed with high-fat diet (HFD) showed that NLRP3 inflammasome was activated in fat, liver, and adipose tissue-derived macrophages (ATMs) through mitochondrial dysfunction and overproduction of ROS, however, importantly, these mechanisms were completely restored by the PPARγ agonist rosiglitazone. CGI-58 is a lipid droplet-associated protein that mediates intracellular fat hydrolysis. Deficiency in this protein leads to the accumulation of cytosolic lipid droplets, hence, limiting the release of FFAs to fuel mitochondrial functions. Consequently, as a result of overnutrition; due to the impaired PPARγ/PGC-1 signaling axis in the mitochondria, significant mtROS is produced, leading to the activation of NLRP3 inflammasome (196).

Nevertheless, different dietary lipids may mediate inflammatory responses in different ways. A study showed that in contrast to the proinflammatory characteristic ω-6-PUFA, ω-3-PUFA-related compounds (such as 3-(S)-HPOTrE and 13-(S)-HOTrE) significantly inhibited the transcription of NLRP3, caspase-1, IL-1β, and IL-18 in LPS-stimulated RAW 264.7 cells and in peritoneal MFs. Interestingly, these effects were reversed when co-incubated with GW9662; a PPAR-γ antagonist, indicating that inactivation of inflammasome is mediated through a PPARγ-dependent pathway by ω-3-PUFA that functions as a natural agonist of PPARγ (197).

The regulatory role of PPARγ on NLRP3 inflammasome activation was also shown in autoinflammatory diseases. Using a model for hyperuricemia-induced kidney injury, pre-treatment with the PPARγ agonist pioglitazone inhibited the MSU crystal-induced NLRP3 protein expression and IL-1β secretion by HK-2 renal tubular cells (198). Later on, an SNP screening of gout patients identified a missense SNP (rs45520937), which causes Arg265Gln (p.R265Q) substitution in the exon 5 of PPARGC1B (PGC-1β), a transcriptional cofactors of PPARγ (199). PBMCs isolated from gout patients carrying this SNP expressed higher level of NLRP3, and the plasma level showed significantly increased IL-1β cytokine level. Transfecting THP-1 cells with plasmids coding for the PGC-1β SNP allele resulted in highly elevated NLRP3 expression and IL-1β cytokine secretion, compared to the wild type PGC-1β transfected samples (200). These results further support the notion that PPARγ/PGC-1 axis has an important modulatory role in IL-1β production in MSU-induced gouty arthritis.

The capability of PPARγ to inhibit NFκB signaling makes PPARγ a potential modulatory factor in the NLRP3 inflammasome-mediated immune responses. For example, in retinal ganglion cells (glia cells), following retinal ischemia/reperfusion, the expression and activation of NLRP3 inflammasome was inhibited by pioglitazone through the inhibition of NFκB and p38 pathway. This neuroprotective effect was reversed by the PPARγ antagonist GW9662 (90, 201).

Similarly, down-regulation of caspase-1, NLRP3, and IL-1β expression was detected following rosiglitazone treatment in irradiation-induced acute intestinal injury as well as in a spinal cord injury (SCI) model, resulting in anti-inflammatory effects and enhanced locomotor recovery (90).

Essentially, PPARγ-mediated NFκB inhibition seems to be an important approach to attenuate inflammatory responses of the intestinal mucosa during colonization with weak pathogens or commensal bacteria. For example, *Bacteroides thetaiotaomicron*, a gram-negative bacterium and one of the most common bacteria found in human gut flora, induces the association of PPARγ and RelA subunit of NFκB, enhancing its nuclear export through a PPARγ-dependent mechanism (202). A similar effect was described in *Neisseria lactamica* (Nlac); an upper respiratory tract (URT) commensal, showing enhanced PPARγ expression and specifically attenuated TLR1/2 signaling by inhibiting NFκB pathway in nasopharyngeal epithelial cells (203). This way, it provides a protective immunity against pathogenic *Neisseria meningitides*, which exclusively inhabits the human upper respiratory nasopharyngeal cavity and occasionally causes epidemic meningitis and a rapidly progressing fatal sepsis. However, importantly, in the URT of elderly mice, a significantly high basal expression of TLR1, NLRP3, and IL-1β was detected, and at the same time, significant overexpression of PPARγ was found. Moreover, no further up-regulation of these factors were detected upon colonization, compared to young adult mice where a marked response was developed. This indicates that mediators of tolerance for commensal bacteria are diminished as part of immunosenescence - in part - through PPARγ-NFκB-NLRP3 pathway. This may explain the increased incidences and susceptibility to respiratory diseases in the elderly (204).

Different subtypes of macrophages have distinct features, mechanisms and dynamics in the secretion of pro-inflammatory cytokines, including IL-1β (205). Furthermore, PPARγ was identified as one of the markers of anti-inflammatory macrophages (M2) (206), and a key driver for alternative MFs polarization (207). This notion is further supported by reports showing that the expression of PPARγ (and PPARβ/δ) is facilitated by STAT6 transcriptional factor, which is a canonical effector of Th2 signaling, and a regulator of mitochondrial oxidative metabolism to fuel M2's activation (208). Furthermore, we reported that during alternative macrophage polarization, activation of IL-4/STAT-6 signaling axis mediated direct transcriptional repression on LPS-induced inflammatory mediators, including NLRP3 and pro-IL-1β in BMDM (209, 210). These results altogether indicate that PPARγ has a combinatorial/coordinating/conductor role in mediating cell-specific (immune) responses.

##### PPARγ and NLRP6

Importantly, studies related to gut homeostasis revealed that not only NLRP3, but also NLRP6 inflammasome is associated with PPARγ-mediated regulatory roles as part of the first line of defense in the innate immune responses. The Flavell group reported, for the first time, that a deficiency of NLRP6 inflammasome in mouse colonic epithelial cells resulted in reduced interleukin IL-18 levels and altered fecal microbiota, explaining the increased susceptibility to colonic inflammation (211), and pointing to NLRP6 inflammasome as an important component of the gut's barrier function. Later, transcript profiling of the mucosal epithelia in developing embryos of rat and sheep revealed that components of NLRP6 inflammasome (NLRP6, ASC, caspase-1), and IL-18, were significantly elevated in the developing intestine, but not in the lung (212) (Figure 5). An *in silico* study of the aligned promoter regions of the human, rat and mouse NLRP6, identified two PPARγ/RXR binding sites in the upstream region that were conserved in the three species. Furthermore, the expression of NLRP6 was highly upregulated in Caco2 human intestinal epithelial cell line, following PPARγ agonist rosiglitazone treatment. PPARγ is known to be involved in intestinal homeostasis, as administration of rosiglitazone to rodents reduced intestinal colitis, and induced the expression of IL-18 receptor on intraepithelial lymphocytes to induce proliferation in response to IL-18. Given that IL-18 is mainly produced by epithelial cells rather than immune cells in the adult rat and mouse intestine, the NLRP6 inflammasome-mediated IL-18 production probably contributes to the homing of lymphocytes to the intestinal mucosa (212, 213).

Under stress (water-avoidance stress) conditions, mice developed enteritis due to inhibition of epithelial NLRP6 expression by the elevated corticotropin-releasing hormone (CRH), while PPARγ agonist rosiglitazone induced NLRP6 expression, and reversed intestinal inflammation (214). Although reports are inconsistent in the intestinal location of NLRP6 expression, studies show that PPARγ activation with its specific agonist has a positive (direct) regulatory effect on NLRP6 expression (215). Furthermore, it highlights the role of (intestinal) PPARγ as an important component of the brain-gut axis.

##### PPARβ/δ

PPARβ/δ is the most ubiquitously expressed of the PPARs. It is activated by a variety of endogenous lipids, including unsaturated fatty acids (FAs), saturated FAs and hydroxyeicosatetraenoic acids. Activation of the receptor induces lipid catabolism through the transcriptional regulation of FA oxidation, mitochondrial biogenesis, and anti-inflammatory response. They also regulate thermogenesis by inducing the expression of UCP in brown adipose tissue and muscle.

Compared to PPARγ and PPARα, the effect of PPARβ/δ on macrophage polarization is controversial, and probably depends on several factors in the microenvironment, the form of the activation, and the origin of the cells. While activation of PPARδ did not influence polarization in human macrophages (216), peritoneal macrophages from PPARβ-deficient mice showed reduced expression of pro-inflammatory genes (217). Nevertheless, in cultured M1 murine MFs, PPAR-β/δ inhibited pro-inflammatory mediators (218), in line with reports showing that synthetic ligands of the receptor exerted anti-inflammatory effects in atherosclerosis, myocardial infarction, acute kidney injury, and lung inflammation (219–221). Furthermore, in murine model of LPS-induced septic shock, PPARβ/δ activation reduced inflammation by inhibiting Akt, STAT3, ERK1/2 and NFκB, iNOS signaling (222).

In association with inflammasomes, Collino et al. described for the first time that in the kidney of high-fructose corn syrup (HFCS-55)-fed mice, increased NLPR3 inflammasome expression, and caspase-1 activation was markedly reduced by the synthetic PPARδ agonist GW0742, which also completely prevented the increase of IL-1β in the serum (223, 224). Similarly, in LPS/PA-treated BMDM, PPARβ/δ activation by GW0742 resulted in a suppressed inflammasome activation (225) (Figure 5).

PPARß/δ was reported as the predominant PPAR subtype in the central nervous system (CNS), and is expressed in all major cell types, including astrocytes, microglia, and neuron (226). While NLRP3 inflammasome-induced neuroinflammation plays a crucial role in dopaminergic neuronal degeneration in Parkinson's disease (PD), agonist treatment of PPARß/δ (GW501516) could suppress NLRP3 inflammasome activation in the midbrain of MPTP-induced mouse model of PD. In this study a decreased astrocyte reaction was reported, while the microglia reaction was not affected by the agonist, indicating that the neuroprotective effects of the PPARß/δ agonist are achieved mainly by targeting astrocytes NLRP3 inflammasome (96).

##### PPARα

PPARα is expressed mainly in higher energy requiring oxidative tissues; such as the skeletal muscle and heart, as well as the liver. PPARα facilitates mitochondrial FFA import and plays a central role in the control of transport, esterification, and mitochondrial β-oxidation of fatty acids, by regulating the expression of the related enzymes. PPARα exerts an important lipid-lowering effect, hence, its down-regulation contributes to heavy lipid accumulation. PPARα was reported to be activated by natural ligands such as fatty acids and their derivatives, as well as by drugs such as the lipid-lowering fibrates (227, 228).

Early reports showed that ligands of PPARα reduced organ injury and inflammation in animal models of shock (229). It was also reported that secretion of acute phase proteins and inflammatory cytokines was clearly decreased upon activation of PPARα in various cells; such as liver, endothelial cells or macrophages, likely through the repression of NFκB pathway (230–233). In line with these findings, PPARα/KO mice showed excessive inflammatory response, greater lung tissue damage, and high mortality rate, compare to the wild type, upon intratracheal administration of *P. aeruginosa*. These effects were meditated by hyper-activation of NFκB pathway, and associated with the up-regulation of NLRP3, and expression of caspase-1 and ASC (89) (Figure 5).

Furthermore, the PPARα agonist fenofibrate, significantly decreased TLR4 and MyD88 expression in a murine model of multiple sclerosis (234), and reversed TXNIP, NLRP3, caspases-1 expression in an *in vivo* study of STZ-induced diabetic mice. In line with these results, in *in vitro* studies, fenofibrate treatment significantly inhibited the LPS-induced IL-1β secretion and ROS production in high glucose-treated endothelial progenitor cells (EPCs). These studies suggested that PPARα may be a good therapeutic candidate to stimulate angiogenesis and accelerate wound healing by deregulating NLRP3 inflammasome activity, and inhibiting IL-1β expression in EPCs of diabetic patients (235).

#### Vitamin D Receptor (VDR)

VDR, upon activation by its ligand (1,25-dihydroxyvitamin D), forms a non-permissive heterodimer with RXR, and binds to the vitamin D-response elements (VDREs) to drive the transcriptional of their target genes. VDRs are also targeted by several post-translational modifications that either enhance or suppress their transcriptional activity. VDR is expressed in a wide range of cells, and has cell-type specific functions, that target several biological processes, including mineral homeostasis, immune responses, cell cycle, and apoptosis (236, 237).

Vitamin D (or cholecalciferol) can be obtained from the diet, or produced by the epidermis following UVB irradiation, after which it is converted to various physiologically active compounds in different parts of the body. Vitamin D is first hydroxylated in the liver to form 25-hydroxyvitamin D_3_ (25-D_3_) or calcidiol; the major circulating vitamin D metabolite in the body. The second hydroxylation occurs mainly in the kidney and peripheral tissues by 1α-hydroxylase, to produce the active vitamin D metabolite 1,25-dihydroxyvitamin D_3_ (1,25-D_3_) or calcitriol (238). A clinical research showed that after supplementation with cholecalciferol, high RNA expression of VDR was found in peripheral blood samples (239). Lack of vitamin D or VDR has been linked to a wide spectrum of health conditions, including autoimmune diseases, chronic inflammation, cancer, and high susceptibility to infections (240, 241). Vitamin D/VDR axis has also an important role in regulating the oxidative stress [reviewed in (238)], and the adaptive immunity; particularly the development and function of T cells [extensively reviewed in (241)]. 1,25-D_3_/VDR signaling mediates the expression of genes involved in antimicrobial responses, including pro-IL-1β. TLR signaling is reported to transcriptionally regulate the expression of VDR and 1α-hydroxylase (236, 237). Furthermore, several studies showed that 1,25-D_3_/VDR signaling has an important role in the regulation of autophagy through different mechanisms, such as inhibition of the PI3K/Akt/mTOR axis, or modulating the expression of autophagy related genes (e.g., ATG16L1, Beclin-1 and PTPN6) (242–245).

First studies regarding the effect VDR on inflammasome function showed that PMA-differentiated THP-1 cells treated with 25-D_3_ or 1,25-D_3_, exhibited increase in IL-1β release. This effect was abolished by caspase-1- and NLRP3 specific inhibitors. Surprisingly, in this study, reduction in NLRP3 mRNA and protein levels were also detected under exposure to 1,25-D_3_ (107).

Nevertheless, in a murine model, VDR was found to inhibit NLRP3 inflammasome both *in vivo* and *in vitro*. VDR-null BMDMs exhibited increase in NLRP3/ASC speck formation in the cytosol. VDR was shown to bind directly to NLRP3 to block BRCC3-mediated NLRP3 deubiquitylation (108) (Figure 4). Notably, licensing of NLRP3 protein through deubiquitylation is a critical step for inflammasome assembly and activation (30). Exposure of BMDMs to 1,25-D_3_ during LPS/Nigericin treatment resulted in inhibition of IL-1β and caspase-1 cleavage, while 1,25-D_3_ had no effect on IL-1β in VDR-null cells. *In vivo*, VDR deficient mice showed a decrease in survival rate, compared to the wild type or NLRP3 null mice (108).

In line with these observations, a report showed that in peritoneal macrophages, 1,25-D_3_-ligated VDR inhibited NLRP3 inflammasome activation induced by various NLRP3 activators (such as ATP, Nig, MSU and alum), and subsequently inhibited caspase-1 activation and IL-1β secretion; while silencing of VDR abolished those effects. Furthermore, activated VDR inhibited NLRB3 binding to NEK7, and blocked NLRP3-mediated ASC oligomerization as well as ROS accumulation, altogether, this disabled the activation of NLRP3 inflammasome. Furthermore, 1,25-D_3−_promoted polyubiquitination of NLRP3 resulted in the degradation and elimination of the protein through autophagy (106).

Human corneal epithelial cells (hCECs) express a wide range of NLRs (246). In dry eye pathogenesis, hyperosmotic stress (the hallmark of the disease) mediated the activation of ROS-NLRP3-IL-1β signaling axis, leading to inflammation of the corneal epithelial. *In vitro*, treatment of hCECs with 1,25-D_3_ suppressed hyperosmotic stress-induced cytotoxicity, NLRP3 inflammasome activation, and IL-1β secretion through VDR activation. Silencing of VDR abolished the effects of 1,25-D_3_ on stress-induced IL-1β secretion and cell survival. These effects are attributed to the ability of 1,25-D_3_ to activate NRF2 antioxidant signaling, by lowering ROS generation, and subsequently abrogating NLRP3 inflammasome activation (104).

Administration of 1,25-D_3_ to a mouse model *of Porphyromonas gingivalis*-induced periodontitis, resulted in the downregulation of NLRP3 inflammasome components in the gingival epithelium. These effects resulted from the ability of 1,25-D_3_ to upregulate the expression of VDR, Aryl hydrocarbon receptor (AhR), and downregulate NF-κB signaling (109). Although, in macrophages, AhR was reported as a negative regulator of NLRP3 inflammasome, by inhibiting NFkB signaling and NLRP3 transcription (247), in order to verify the direct mechanism of AhR on inflammasome regulation in the gingival epithelium, additional studies are required. Furthermore, treatment of MRL/lpr mice (a model of SLE) with VDR agonist paricalcitol decreased the pathogenesis of lupus nephritis, through inhibiting NFκB/NLRP3/caspase-1/IL-1β/IL-18 axis in the renal tissue. Mechanistically, VDR was shown to competitively bind importin 4 and inhibit importin 4-mediated NFκB nuclear translocation (105).

These data suggest that vitamin D/VDR axis is an important regulator of inflammasome function. Nevertheless, as VDR transcriptionally regulates more than 900 genes, the results obtained from different sources, or based on VDR deletion studies, should be supported with further detailed studies.

#### Retinoic Acid Receptor (RAR)

RARs (RARα, β, γ) are ligand dependent transcription factors that function as obligate heterodimers with RXRs. RAR/RXR complex is a non-permissive heterodimer, requiring a ligated RAR partner for activation, while in the absence of ligands, the complex mediates transcriptional repression. As an additional level of regulation, their function can be modulated by various post-translational modifications (PTM). Among several identified retinoic acid derivatives, all-trans retinoic acid (ATRA) is the predominant isoform in the body, and drives the majority of the biological functions of vitamin A (248). Although RARα has ubiquitous expression, the other isoforms exhibit distinct and tissue-specific expression patterns (249).

Activated RARs may also induce rapid non-transcriptional effects through interaction with several kinase cascades. RAR-mediated signaling pathways have pleiotropic functions, and are involved in several critical biological processes; such as cell proliferation, differentiation, metabolism, tumorigenesis, hematopoiesis and immune responses (248, 250, 251). Regarding immune responses, RARs play a central role in modulating the differentiation and function of a spectrum of innate and adaptive immune cells, and any defect in these pathways, or absence of their agonists (e.g., vitamin A deficiency) may be associated with several metabolic and immune disorders [extensively reviewed in (252–255)].

**Figure 6:**
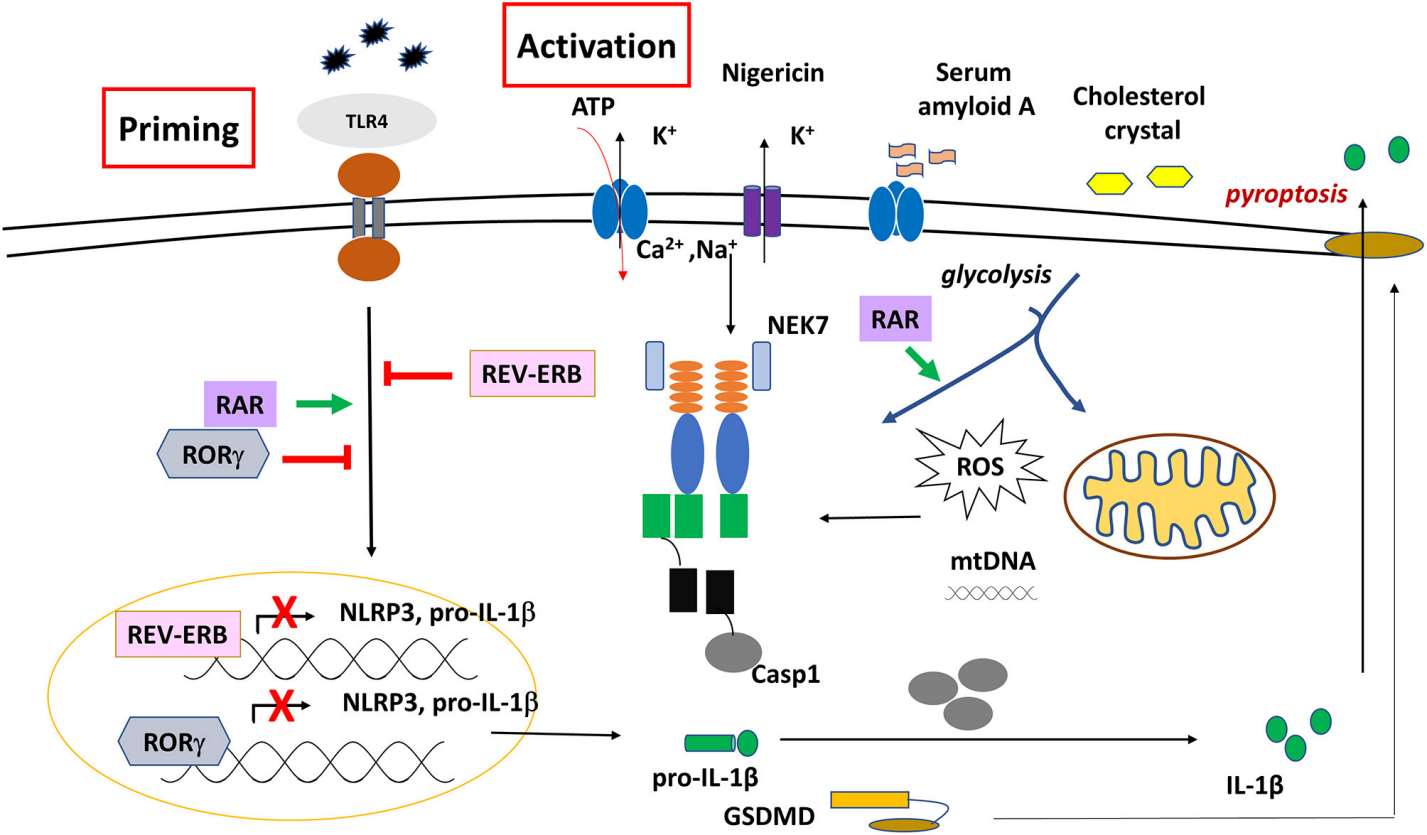
Regulatory role of RAR, REV-ERB, and RORγ on NLRP3 inflammasome functions.

Similar to other nuclear receptors, activation of RAR may lead to various effects, depending on the particular cell, and the microenvironment. ATRA was reported to mediate caspase-1 activation in cervical carcinoma cells through IRF-1/STAT1 axis, which suppressed the growth of the cells (256). ATRA has the ability to promote autophagy following intracellular bacterial infection, through interaction with canonical PI3 kinase, Beclin-1, and TBK1 pathway of autophagy (257). Modulation of IL-1β secretion by ATRA has been reported for various myeloid, and non-myeloid cells, such as macrophages and mast cells. Studying THP-1 cell line, it was shown that ATRA augmented IL-1β secretion upon *Bacillus subtilis* flagellin challenge in RAR/RXR-dependent manner. The effect of ATRA on IL-1β secretion was due to the ability of ATRA to enhance the NFκB pathway (99) (Figure 6). Recently, we reported that ATRA treatment enhanced basal expression of NLRP3 in human monocytes and macrophages. In LPS-activated macrophages, ATRA upregulated the expression of NLRP3 and pro-IL-1β, furthermore, it enhanced caspase-1 activation and NLRP3 inflammasome-induced IL-1β secretion. This was attributed to the ability of ATRA to enhance the signal transduction pathways (like NFκB, p38, ERK), and to inhibit AKT/mTOR signaling. Also, it was shown that ATRA enhanced the LPS-induced glycolytic activity of the cells, in part, by inducing hexokinase-2 expression, which also participated in the enhanced NLRP3 inflammasome activation (97).

In contrast, in a murine model of alcohol-induced toxicity, an *in vivo* study showed that ATRA induced the downregulation of NLRP3, pro-caspase-1 and pro-IL-1β mRNAs in the brain tissue, upon endotoxin treatment. This report demonstrated that while chronic alcohol exposure increased the intestinal permeability for endotoxins, and subsequently elevated the ROS level in the circulation, ATRA developed a protective effect by inhibiting these mechanisms (98).

In mouse intestine epithelial cells, knockdown of RARα (the most abundant isoform in this cell type) correlated with down-regulation of IL-18, which was associated with the increase in bacterial burden (258). It is suggested that IL-18; produced by intestinal epithelial cells, is responsible for gut homeostasis and T lymphocyte homing, thus, the link between RAR signaling and inflammasome-mediated IL-18 secretion may warrant further investigation.

#### REV-ERB and RORs

Retinoic acid receptor-related orphan receptors (RORs) have several isoforms (RORα 1-4, RORβ, RORγ1,2 or t), and mainly recognize their response elements as monomers. Expression of these isoforms is controlled by the variable promoter usage and alternative splicing, and exhibits tissue-specificity (259, 260). For instance, RORγt is specifically expressed in immune cells, and is considered a key transcription factor for Th17 cell development. Indeed, Th17 cells are implicated in the development of several autoimmune diseases (e.g., autoimmune arthritis), and mice lacking RORα and RORγ exhibit absence of Th17, and are susceptible to developing autoimmune disorders (260–262).

Unlike the other nuclear receptors, REV-ERBs (REV-ERBα and REV-ERBβ) bind to their response elements as a monomer or homodimer. REV-ERBs lack the AF2 transactivation domain which is required for binding co-activators and transcriptional activation. Hence, as they recruit the co-repressor (NCoR) and histone deacetylase 3 (HDAC3) complexes to their target genes, they are considered as transcriptional repressors (261, 263). Transcriptionally, REV-ERBs is rhythmically regulated by the core circadian clock complex (BMAL1/CLOCK), and in turn, as part of the feedback loop of the circadian clock genes, REV-ERBs directly represses the transcription of *BMAL1*. Furthermore, REV-ERBs compete with RORs (α, β, γ) nuclear receptors (described below) to bind to the promoter of *BMAL1*, leading to the suppression of ROR-induced *BMAL1* expression. Aberrant regulation of either receptors is associated with alteration in the circadian rhythm (261, 264).

Indeed, both REV-ERBs and RORs share the same DNA response elements, target genes, and usually co-expressed in the same tissues. Beside the regulation of circadian rhythm, these receptors have also overlapping functions in the regulation of lipid homeostasis, metabolism and immune response (261, 265). However, they identify different ligands, for example heme functions as an endogenous ligand for REV-ERBs, while sterols, oxysterols and cholesterol derivatives act as ligands for RORs. Multifaceted molecular mechanisms of interaction and crosstalk between the circadian clock and immune functions including REV-ERBs and RORs are reviewed elsewhere (266, 267). Also, oscillatory expression of NLRP3 inflammasome-related genes or -activity had been reported in several studies (101, 268).

Dysregulation in the expression of circadian clock genes including Rev-erbα and Rev-erbβ has been reported in DSS-induced colitis mouse model. Disruption of circadian clock or deletion of Rev-erbα aggravates colitis conditions, while opposite effects are seen in response to SR9009. Of note, SR9009 was originally designed as an agonist for REV-ERB, however, recently its REV-ERB-independent effects has been also reported (269). Nevertheless, using REV-ERBα –/– mice, increased NLRP3 and IL-1β expression was detected, and REV-ERBα was linked to experimental colitis through its ability to repress the expression of *p65* subunit of NFkB, which subsequently leads to the suppression of NLRP3 inflammasome at the priming level (100). However, in experimental colitis, recent report has indicated that NF-κB signaling mediates REV-ERBα expression through Lnc-UC (long non-coding RNA), as a self-healing mechanism to restrain the inflammation (270) (Figure 6).

CHIP analysis of human and mouse macrophages revealed a direct binding of REV-ERBα to the promoters of NLRP3 and IL-1β, resulting in a repressed expression. At pathophysiological level REV-ERBα deficient mice displayed increased susceptibility to peritonitis and LPS/D-Galactosamine-induced fulminant hepatitis, whereas activation of REV-ERBα restrained the pathological conditions in an NLRP3 inflammasome-dependent manner (101). However, REV-ERBα; expressed in human macrophages, transcriptionally repressed IL-10 expression by recruiting the co-repressor NCoR and HDAC3 to its promoter region as part of antimicrobial defense mechanism. Interestingly, in mouse, the REV-ERBα response element motif in IL-10 promoter is disrupted (271).

Similarly, RORγ knock out or RORγ inverse agonists (SR1555, SR2211) diminished NLRP3 inflammasome activation in BMDMs. Indeed, RORγ regulates NLRP3 inflammasome by direct binding to NLRP3 and IL-1β promoters in multiple putative sites. Notably, the same sites were also reported to be recognized by REV-ERBα. *In vivo*, RORγ inverse agonists render protective effects against sepsis and fulminant hepatitis in mouse models mediated by suppression of NLRP3 inflammasome (101, 102). In contrast, dysfunctional RORα (RORα^sg/sg^) in septic mice exhibits high levels of NF-κB signaling, active caspase-1, and IL-1β production (272). In addition, RORα recruits the corepressor HDAC3 to NF-κB target promoters, which leads to transcriptional repression of the inflammatory genes (including IL-1β) in DSS-induced colitis mouse model (273) (Figure 6).

Altogether, given the fact that irregularity in the circadian oscillation is associated with several pathological conditions, these data suggest an important role for REV-ERBs and RORs in NLRP3 inflammasome regulation, and suggests an association between inflammatory response and circadian rhythm.

## Concluding Remarks and Future Perspectives

This review attempts to draw the attention to nuclear receptors as important modulators of NLRP3 inflammasome function. Nuclear receptors are distributed throughout most tissues and cells, and participate in a wide range of physiological and cellular processes by sensing endogenous or exogenous lipid compounds. Importantly, many of these lipids, their metabolites, or the products of nuclear receptors' target genes can be related directly or indirectly to NLRP3 inflammasome function. Furthermore, NLRP3 is the target of many drug studies as it regulates acute and chronic inflammatory diseases. Although we highlighted a number of mechanisms by which nuclear receptors may regulate the activity of the NLRP3 inflammasome, further detailed studies are required to get a better understanding of the molecular mechanisms that associates the two very complex field. The outcome of nuclear receptors activity in many cases seems to be tissue specific, and dependent on the particular microenvironment and conditions. The fact that nuclear receptors are at the crossroad of metabolism and immune responses, makes them ideal potential targets in therapeutic approaches, where the manipulation of NLRP3 inflammasome-mediated cytokine secretion is the aim. However, due to the coordinating role of nuclear receptors in lipid and glucose metabolism, therapeutic usage should be well-designed, and cautiously targeted. Altogether, it requires further investigations to understand the complex cooperation of nuclear receptors with Nod-like receptors, but without doubt, it holds exciting outcomes in the future.

## Author Contributions

AA: writing—original draft preparation. SB: writing—original draft preparation, review and editing, supervision. All authors have read and agreed to the published version of the manuscript.

## Conflict of Interest

The authors declare that the research was conducted in the absence of any commercial or financial relationships that could be construed as a potential conflict of interest.

## Abbreviations

1,25-D_3_: 1,25-dihydroxyvitamin D_3_
MPTP: 1- methyl-4-phenyl-1,2, 3, 6-tetrahydropyridine
AKR1B7: Aldo-keto reductase family 1, member B7
ASC: Apoptosis-associated speck-like protein
ATRA: All-Trans Retinoic Acid
BMDMs: Bone marrow-derived macrophages
CHOP: C/EBP homologous protein
CAR: Constitutive androstane receptor
DAMPs: Damage-associated molecular patterns
ER: Endoplasmic reticulum
FXR: Farnesoid X receptor
GPBAR 1 TGR5G: protein-coupled bile acid receptor 1
HAMPs: Homeostasis-altering molecular processes
LXR: Liver X receptor
BRCC36: Lys-63-specific deubiquitinase
mtROS: Mitochondrial reactive oxygen species
NLRs: NOD-like receptors
NKC1: Non-catalytic region of tyrosine kinase adaptor protein 1
NAFLD: Nonalcoholic fatty liver disease
PD: Parkinson's disease
PRRs: Pattern recognition receptors
PPARs: peroxisome proliferator-activated receptors
PPE: Porcine pancreas elastase
PKC: Protein kinase C
RAR: Retinoic acid receptor
RXR: Retinoid X receptor
ROR: Retinoic acid receptor-related orphan receptor
PERK: RNA-like endoplasmic reticulum kinase
SREBF1: Sterol regulatory element-binding transcription factor 1
SHP: Small heterodimer partner, NR0B2
TLRs: Toll-like receptors
VDR: Vitamin D Receptor.
